# Monitoring the United Nation’s Convention on the Rights of Persons with Disabilities: data and the International Classification of Functioning, Disability and Health

**DOI:** 10.1186/1471-2458-11-S4-S8

**Published:** 2011-05-31

**Authors:** Jerome E Bickenbach

**Affiliations:** 1 Department of Health Sciences and Health Policy, University of Lucerne; 2 Swiss Paraplegic Research (SPF), Nottwil, Switzerland

## Abstract

This paper approaches the general issue of the complex challenges in the relationship between those who generate data -- researchers, scientists, and state statistical offices -- and those who use data -- researchers and policy-makers -- in light of the more specific policy challenges created by the monitoring requirement of the United Nation’s Convention on the Rights of Persons with Disabilities (CRPD: Article 33). International Conventions and Treaties standardly suffer from being persistently ineffectual primarily because of the absence of implementation mechanisms. The CRPD, by contrast, explicitly requires State Parties who have ratified it to institute data generation and monitoring mechanisms for its implementation. This paper argues that WHO’s International Classification of Functioning, Disability and Health (ICF) can be brought into the service of the CRPD data generation and monitoring mandate, both in the shaping of relevant data streams and in the creation of relevant indicators, and concludes by reviewing the challenges that remain.

## Background

This paper is about the policy challenges created by the monitoring requirement of the United Nation’s Convention on the Rights of Persons with Disabilities (CRPD) [1]. These challenges are part of a much larger issue concerning the relationship between data generators (scientists, researchers, and state statistical offices that produce, analyse, collate, store and disseminate information) and data users (again researchers, but also other agencies of the state mandated or required to use population-based data in various ways). Simplistically, it is a matter of building bridges.

In the area of disability policy generally, because of the long history of the ‘medicalization’ of disability and the relatively recent ‘social’ understanding of disability, the primary bridge that needs to be constructed and maintained at the governmental level is that between health ministries and social ministries – both of which have jurisdiction over disability issues, but rarely communicate. The primary purpose of this paper is related to this problem, but focuses on the specific challenge of implementing the CRPD by means of an evidence-based monitoring mechanism – a challenge, which I argue, might best be met by building a bridge between the World Health Organization’s International Classification of Functioning, Disability and Health (ICF) [2] and the CRPD. But, as mentioned, there is another, and conceptually more fundamental bridge that needs to be built: that between policy data users and governmental statistical offices, data generators.

For historical and political reasons, disability policy data users and governmental data generators have a long tradition of antagonism. Data users, the policy people, say that the data they get from population health survey, longitudinal surveys, censuses, even administrative records, tend to reflect the principle of ‘counting what is easy to count’

For their part, statistical offices insist that the data they are asked to provide is not scientifically valid or reliable data, but political-motivated information serving a thinly disguised political agenda.

The gap is all the more difficult to bridge because it is a manifestation of a discontinuity between science and politics, or as it is sometimes put, between facts and values. Data generators who use sophisticated survey methodologies or information technology models to construct data collection instruments feel themselves firmly on the side of science, whereas policy analysts associate themselves more closely with political and social agendas that are, and should be, value-based.

The irony is that, on closer inspection, data-generating scientists cannot function without values, in particular the values of validity, reliability and comparability, whereas policy analysts who remove themselves from the world of concrete and reliable facts are simply not connecting with the world they must describe and influence. The ICF, arguably, avoids both mistakes by creating a platform for disability policy, infused with the values of the CRPD, which is at the same time the basis for respectable and sound science. To make this claim plausible, I need to set the stage, both in terms of ICF and its characteristics, and in terms of the monitoring requirements of the CRPD.

### ICF and CRPD

**Figure 1:**
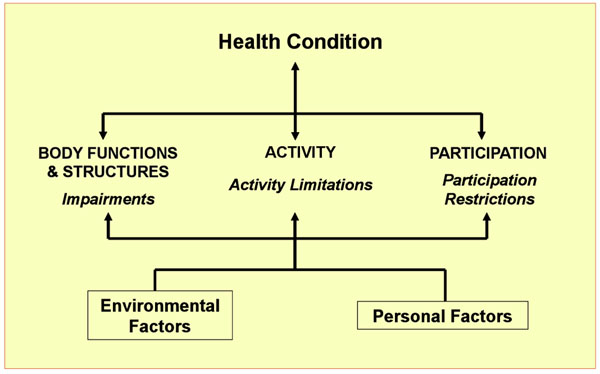
WHO 2001

ICF provides both a model of functioning and disability and a set of classifications for describing these phenomena in detail. ICF understands these phenomena as outcomes of an interaction between an underlying health condition (disease, disorder or injury) and the full range of environmental factors (physical, human-built, social and attitudinal) and personal factors. Figure 1 is a diagram of this familiar model.

The four classifications found in the ICF – Body Functions, Body Structures, Activity and Participation, and Environmental Factors – are codified and operational, exhaustive and -- as between these four dimensions -- mutually exclusive lists of categories that can be used to validly and consistently describe aspects of functioning and disability, generally or in detail, for individuals in a clinical setting, or populations through surveys, questionnaires and censuses.

Since 2001, when it was endorsed by the 191 countries of the WHO World Health Assembly, ICF has been used in countless applications to provide a consistent and comparable, international language of the lived experience of health to generate internationally comparable health and disability data [3]. ICF applications at the clinical and research levels have predominated, but, increasingly, it has also been applied in the policy arena, and in particular for eligibility determination.

At the same time, the period of time during which the ICF was developed, endorsed and began to be implemented overlaps with a period of development and innovation in another area of concern to persons with disabilities: the recognition of their basic human rights.

In 1993, the *Standard Rules on the Equalization of Opportunities for Persons with Disabilities*[4] was proclaimed in effect and welcomed by disability NGOs and disability advocates around the world. The *Standard Rules* is a document of extraordinary scope and vision built on two interlocking, foundational concepts, that of ‘equalization of opportunities’ and the ‘principle of equal rights’. Although the *Standard Rules* provided for a monitoring process for effective implementation of the 22 Rules – namely the creation of an office of a Special Rapporteur and a voluntary and self-funded panel of experts from international disability organizations to advise the Rapporteur – the results after a decade have been somewhat disappointing. The underlying problem was that the *Standard Rules* has no enforcement mechanism other than persuasion and voluntary compliance. In addition, in light of the absence of clear indicators of progress and a mechanism for collecting and analyzing data relevant to these indicators, there is no evidence-based way of identifying, let alone assessing, implementation.

On December 13, 2005 in New York, the United Nations Convention on the Rights of Persons with Disabilities was passed by the UN General Assembly. Since that date, the CRPD has been signed by most of the countries in the world, including, for the first time, the European Commission. Individual State Parties which have both signed and ratified the CRPD [currently (17.1.2011) there are 147 signatories and 97 ratifications] are obliged to implement the Convention in their country and to monitor the progress of implementation. An Optional Protocol provides for the additional, and important, right of individuals or groups to petition the appropriate *Convention* body to review the implementation progress of a country.

Although in the underlying philosophy and normative content the CRPD is very similar to the *Standard Rules* – sharing in particular what is often called the ‘rights approach to disability’ and a commitment to a process of change toward a more inclusive society –, in one important respect they are totally different. In a word, the CRPD is a true *instrument of international law*, since it is a treaty the ratification of which has clear legal consequences. Legal scholars are suggesting that this fact will have direct impact on the policy arrangements within States Parties [5-8].

## CRPD: scope and monitoring

The scope of the CRPD includes all areas of human experience, consistent with its underlying rationale, as expressed in the Preamble:

*Reaffirming the universality*, *indivisibility*, *interdependence and interrelatedness of all human rights and fundamental freedoms and the need for persons with disabilities to be guaranteed their full enjoyment without discrimination…*

The first four Articles of the CRPD set out the purpose, principles, obligations, definitions and overarching principles (or rather, categories of rights: dignity, non-discrimination, full and effective participation, respect for difference, equality of opportunity, accessibility), whereas the five Articles at the end describe the monitoring and reporting process (Articles 32-36). Between these bookends are 27 substantive Articles describing areas of human life or experience that fall into two groups: Articles that state general principles of equality and inclusion (without specifying goals or objectives), and those that identify substantive areas of interest and specify goals and sub-goals. The statements of principle, though important elements of the CRPD, are primarily interpretative principles. Some of the basic rights found in the CRPD are in Table 1.

**Table 1:** Some of the protected rights in the CRPD

Convention on the Rights of Persons with Disabilities Some of the protected rights
Article 9 Accessibility

Article 11 Situations of risk and humanitarian emergencies

Article 12 Equal recognition before the law

Article 13 Access to justice

Article 15 Freedom from torture, cruelÂ…degrading treatment or punishment

Article 16 Freedom from exploitation, violence and abuse

Article 17 Protecting the integrity of the person

Article 18 Liberty of movement and nationality

Article 19 Living independently and being included in the community

Article 20 Personal mobility

Article 21 Freedom of expression and opinion, and access to information

Article 22 Respect for privacy

Article 23 Respect for home and the family

Article 24 Education

Article 25 Health

Article 26 Habilitation and rehabilitation

Article 27 Work and employment

Article 28 Adequate standard of living and social protection

Article 29 Participation in political and public life

Article 30 Participation in cultural life, recreation, leisure and sport

The core of the monitoring provisions of the CRPD are Articles 31 and 33.

In brief, what they require of States is that they:

• collect “appropriate information” to enable States “to formulate and implement policies to give effect to“ the CRPD (Article 31);

• designate a focal point within government on implementation; and

• designate a mechanism “to promote, protect and monitor implementation of the present Convention (Article 33).

Nothing more is said about what this monitoring mechanism should consist of, how relevant information should be collected or processed, or how the relevant information should feed into the monitoring process. That is left to the discretion of each country, although it is plainly understood that both the mechanism and the data collected should be subject to the highest scientific standards appropriate to an evidence-informed monitoring process.

## Components of monitoring

A monitoring mechanism for the implementation of any set of rules or prescriptions for change (such as those found in a human rights instrument) should be clearly distinguished from the components of the monitoring process. The mechanism may be voluntary or mandatory, it may use the services of a Special Rapporteur or rely on a committee of experts, its products may be reports that are critical of a countries efforts or, in the extreme, involve enforcement sanctions for failure to meet specific goals. Whatever the mechanism, however, there are five key elements of the process of monitoring: rights, goals, targets, indicators, and data sources [9,10].

### Rights

In the case of human rights instruments, stated rights create duties or obligations on one party (typically but not necessarily, the state) and entitlements or rights on another (the citizen or some other beneficiary). Rights are pure form of normatively, which is why Gerald Quinn has identified the rights in the CRPD as forming the ‘moral compass’ of disability policy reform [5].

### Goals

From the monitoring perspective, however, what is important about rights – why they can serve as a moral compass – is that they determine policy goals. If, as sometimes happens, statements of human rights are so vague or abstract that they do not determine any goals (e.g. ’Everyone has the right to proper treatment by the state’ or ‘Everyone has the right to be treated correctly’), or do not clearly determine specific goals (e.g. ’People have the right to dignity’), then statements of rights are not operationalizable into goals and, political rhetoric aside, they are of little use to people. In a recent international human rights document, the rights are implicit and unstated, so that the document itself speaks entirely in terms of goals. The Millennium Development Goals (MDGs) are eight general, international, social objectives that respond to what has been agreed to be the world's primary development challenges, in light of the values and principles stated in the Millennium Declaration adopted in 2000 [11]. To illustrate, the first goal of the MDGs (poverty eradication) is set out in Table 2.

**Table 2:** Goal 1 of the Millennium Development Goals

Millennium Development Goals (MDGs)	
**Goals and Targets (from the Millennium Declaration)**	**Indicators for monitoring progress**

**Goal 1: Eradicate extreme poverty and hunger**	

**Target 1:** Halve, between 1990 and 2015, the proportion of people whose income is less than one dollar a day	**1**. Proportion of population below $1 (PPP) per day **2**. Poverty gap ratio [incidence x depth of poverty] **3**. Share of poorest quintile in national consumption

**Target 2:** Halve, between 1990 and 2015, the proportion of people who suffer from hunger	**4.**Prevalence of underweight children under five years of age **5.**Proportion of population below minimum level of dietary energy consumption

In the case of the CRPD, like the 1966 International Covenant on Economic, Social and Cultural Rights (ICESCR) [12] before it, the CRPD goals and sub-goals often need to be drawn out of the wording of each Article. This inevitably leads to interpretative issues and the importance of being faithful to the text of the CRPD. The alternative of explicitly stating the goals of the CRPD -- as the MDG does -- would have, arguably, limited the impact of the CRPD.

### Targets

Targets are qualitative or quantitative operationalizations of goals; they provide a concrete description of the content of the goal and specify details about the precise social commitment the goal creates. It is quite possible that a single goal generates several targets that may overlap in practice. But the essential feature of targets is that they specify the kind, degree or extent of achievement of a goal, which, optimally, is expressed quantitatively. Table 2, once again, shows the quantitatively-expressed and highly specific targets associated with MDG Goal 1: “Halve, between 1990 and 2015, the proportion of people whose income is less than one dollar a day“.

Targets are, after the rights themselves, the most important components of the monitoring process, and the most challenging to develop as they involve a delicate balance: targets must be both forward-looking and progressive, they must move us in the direction that the rights proclaim and the goals point us to. At the same time, they cannot be utopian or realistically unachievable. One tactic to secure this balance between progressive and realistic is to make targets time-limited (“Halve, between 1990 and 2015…“) in order to facilitate coordinated action and mobilize both political and economic resources, while at the same time keeping awareness of the issue prominently on the political agenda in order to create a sense of urgency. Targets, if well crafted, should walk a tightrope: challenging yet feasible, immediate yet not so demanding as to generate skepticism or fatalism in policy makers or advocates, and progressively idealistic without being utopian.

Targets may be *absolute* (e.g. the MDG target for material mortality ratio states: “Reduce by three-quarters, between 1990 and 2015, the maternal mortality ratio”), in which an explicit, measurable target is specified using a measurement metric that is scientifically acceptable and politically understandable. Targets may also be *relative*, in the sense that they set a target in terms of the level of progressive achievement of those countries which have achieved the most: “Achieve the maternal mortality ratio that is within the top 10 countries of the world.” Both kinds of targets have their virtues and problems. An absolute target is scientifically measurable; a relative target is too easy to politically manipulate. In the CRPD, no targets as such are given. It might not be inappropriate for the World Health Organization or some other United Nations agency, under the rubric of a monitoring exercise, to specify targets for its Member States; it is perfectly appropriate for it to specify its own targets for its own. Article 32 - International cooperation implies that the UN specialty agencies (WHO, ILO, and UNESCO) may be called upon to provide technical assistance in the development of the components of a monitor mechanism.

### Indicators

These are variables, in the statistical sense, that can be used to identify or measure change over time (once again, Table 2 shows the indicators recommended for the MDG targets under Goal 1). For obvious reasons, it is best that these indicators be standardized and international [13]. Indicators often follow nearly automatically from the wording of a target. For example, MDG Target 5 is: ”Reduce by two-thirds, between 1990 and 2015, the under-five mortality rate, the obvious indicator is under-five mortality rate” – obviously the indicator is mortality, measured by rate of incidence of death [14]. The bulk of recent work on human rights indicators has been pursuant to ICESCR [15], in which indicators are developed directly from the higher level goals. Although this procedure and the ICESCR generally are good models for the development of CRPD indicators, there is a danger in pursuing this tactic since, in effect, the indicator selected would imply that the hoped for outcome is not a single, explicit target but rather a range or spectrum of potential targets, which might diffuse the political will to achieve the result specified by the goal, or background right [15].

### Data source

The final component of the monitoring matrix is the stream of valid and reliable data that is relevant to the indicator that has been selected to operationalize the right in terms of goals and targets. Article 31 of the CRPD requires States to collect information that enables them to formulate and implement CRPD-consistent policies that is “disaggregated”. It does no good, in other words, to collect data on employment rates if ‘disability’ is not used as a demographic variable so that a comparison can be made between overall employment rates and rates for persons with disabilities.

### Challenges of monitoring the CRPD

Despite its policy potential and the breadth of its coverage, the CRPD creates considerable challenges to the State, which is required by ratifying the convention to create an implementation monitoring mechanism. These challenges are both political and scientific. Even an enthusiastic political acceptance of rights and goals may not be easily translated into an equally enthusiastic acceptance of targets.

Compared to the political challenges, the scientific challenges may seem almost technical and unimportant, but that is far from being so, as the experience of the ICESCR has suggested [16,17]. This in turn requires a bridge to be built between the science of indicator development and data generation, and the values inherent in the goals and rights of the CRPD. At the end of the day, only those targets that can be politically endorsed will form part of a State’s monitoring mechanism. Given the substantial range of rights and underlying goals expressed or implied in the CRPD, it is a challenge merely to devise a collection of targets that span the full scope of the CRPD. Recalling the selection of substantive provisions set out in Table 1, targets need to be established for a range of policy areas. At the same time and in addition, each of these targets has to be feasible, yet progressive, achievable but not trivial, and finally, measurable. A daunting challenge indeed.

### The potential role of ICF in convention monitoring

I would like to suggest that the ICF can play two very important ‘bridging’ roles in the monitoring process, one scientifically technical, the other more political but, from the standpoint of the future of the realization of rights for persons with disabilities, essential.

Technically, the ICF is the only world standard, proven to be valid and reliable, that is available for disability data collection and management. Indeed, the ICF offers a classification scheme based on a hierarchically arranged coding structure. In part as well, the ICF provides an informational model of functioning and disability (see Figure 1 above) that is true to the complex character of disability phenomena. *Prima facie*, then, where monitoring requires data collection by means of a survey, questionnaire, administrative record or some other mechanism, ICF offers the prospect of coordinating internationally comparable disability data relevant to the CRPD monitoring process.

This remark is, of course, purely conceptual and there is much work ahead to ground this conjecture empirically. But the conjuncture is plausible. It is a relatively easy exercise to crosswalk the subject matter of the rights set out in the CRPD with ICF participation domains, at least at the Chapter level (see Table 3).

**Table 3:** CRPD rights and ICF categories compared

Convention on the Rights of Persons with Disabilities	ICF Participation Domains
Article 19 Living independently and being included in the community	Chapter 5 Self-care Chapter 9 Community, social and civil life

Article 20 Personal mobility	Chapter 4 Mobility

Article 21 Freedom of expression and opinion, and access to information	Chapter 3 Communication

Article 23 Respect for home and the family	Chapter 7 Interpersonal interactions and relationships: Particular interpersonal relationships

Article 24 Education	Chapter 8 Major life areas: Education

Article 25 Health	Chapter 6 Domestic life

Article 26 Habilitation and rehabilitation	Chapter 6 Domestic life

Article 27 Work and employment	Chapter 8 Major life areas: Work and employment

Article 28 Adequate standard of living and social protection	Chapter 8 Major life areas: Economic life

Article 29 Participation in political and public life	Chapter 9 Community, social and civil life

Article 30 Participation in cultural life, recreation, leisure and sport	Chapter 9 Community, social and civil life

But, more particularly, in many instances there are more granular links possible. As an example, consider the rights under Article 26 of CRPD with respect to the provision of habilitation and rehabilitation services.

Like many of the Articles in CRPD, Article 26 is structured in a way to highlight both the underlying rights (“…to enable persons with disabilities to attain and maintain maximum independence, full physical, mental, social and vocational ability, and full inclusion and participation in all aspects of life”) and goals (“…shall organize, strengthen and extend comprehensive habilitation and rehabilitation services and programmes, particularly in the areas of health, employment, education and social services, in such a way that these services and programmes”) as well as sub-goals (“Begin at the earliest possible stage, and are based on the multidisciplinary assessment of individual needs and strengths”). Targets, however, for reasons already mentioned, are not provided.

Goals and specific targets relevant to these goals lend themselves naturally to a data collection instrument such as a questionnaire or household survey, in which respondents are asked, for example, "Do you receive the rehabilitation services you feel you require?". The data collected from this questionnaire would need to be compared and collated with other related data from a variety of sources, clinical and administrative records, population health surveys among others. To compare these data, however, it is essential that the relevant monitoring category (‘rehabilitation services’ in this instance) be related across data sources. And this is precisely what ICF provides the basic mechanism for doing so in its classifications and coding system (in this instance, the relevant ICF code is e5800).

This application of the ICF is more significant than it might at first appear. Unless it is possible to compare data across data collection instruments and modalities, it will not be possible to construct a summary measure relevant to the target, whether absolute or relative, associated with these CRPD rights. More importantly for the CRPD monitoring exercise, without data comparability, the summary measures generated by different countries would not themselves be comparable, and no sensible judgment about the extent or degree of relative implementation of Article 26 could be made. Thus, in this simple way, the ICF deals with one of the most significant scientific challenges to an evidence-based monitoring process – whatever mechanism chosen.

The broader application of the ICF would need to involve the full range of targets, indicators and data sources. As a trial run and proof of concept, consider the matrix in Table 4. In this example, the targets have been invented for illustration; in the actual case they would be the result of a political debate at the national level. The indicators here are also suggestions for illustration. That said, it is fairly clear what the minimal role of the ICF could be in this process: that of bridging in operational terms the indicators and the existing data sources available at the country level. In this manner, it would be possible to determine not only how best to use existing data, but also where data gaps relevant to the selected indicators exist. These gaps would be expressed as ICF categories of disability for which no appropriate national level data exists. Were the whole process to be implemented – and all of the cells in the matrix filled – then not only it would get the country be able to quantitatively assess its level and rate of implementation over time, but, at any particular time, it would be able to compare its progress with other countries which operated in terms of the same matrix, irrespective of the targets they select (as long as these targets are themselves comparable).

**Table 4:** A CRPD monitoring matrix

Convention GOAL	TARGET	INDICATORS	ICF code	Data Source
**Article 26 Habilitation and rehabilitation**				
*1. States Parties shall take effective and appropriate measures*, *including through peer support*, *to enable persons with disabilities to attain and maintain maximum independence*, *full physical*, *mental*, *social and vocational ability*, *and full inclusion and participation in all aspects of life. To that end*, *States Parties shall organize*, *strengthen and extend comprehensive habilitation and rehabilitation services and programmes*, *particularly in the areas of health*, *employment*, *education and social services*, *in such a way that these services and programmes.*	Provide comprehensive habilitation and rehabilitation services and programmes (health, employment, education and social services), to persons with disabilities that is: • based on standards of multidisciplinary assessment of individual needs and strengths • based on standards of participation and community inclusion • voluntary • available and community-based	Proportion of persons with disability in need of rehabilitation services, who are receiving them.	↔	**Household and facility surveys**
		Proportion of population of persons with disability in rural areas receiving necessary rehabilitation services.	↔	**Administrative records**

## Conclusion

These suggested uses of ICF for CRPD monitoring are obviously preliminary. But they are also intuitively robust: on the assumption that the ICF classification system is, as it purports to be, exhaustive and consistent, then there is reason to be confident in its potential linking function between indicator data requirements and existing data sources.

The ever-expanding literature on ICF implementation suggests that the ICF model of functioning and disability potentially offers more than merely a guide for structuring disability data. But its data structuring is essential for a scientifically sound CRPD monitoring process. At the same time, the ICF exemplifies in its model of functioning and disability the so-called ‘biopsychosocial concept of disability’, in which disability is a multi-dimensional concept that constitutes the outcome of interactions between intrinsic features of the person and the person’s physical, built, attitudinal and social and political environment. This conception is fully aligned with the so-called ‘rights approach to disability’, in which the focus in disability policy is shifted from the ‘personal misfortune’ of a decrement in health to a fully contextualized lived experience of functioning as effected, positively or negatively, by the individual physical and social context. The ICF is at once both a scientific and a rights-based instrument [18,19].

Thus ICF is, in effect, a potential bridge, not merely between indicators and data sources – which is, in practical terms, an essential facilitator of a scientifically respectable monitoring process – but also a bridge between scientific values and the political and social values expressed in the rights in the CRPD.

## Competing interests

The authors declare that they have no competing interests.
